# Every Landsman's Duty and Opportunity

**Published:** 1917-07-14

**Authors:** 


### KING GEORGE'S FUND FOR SAILORS.

### Every Landsman's Duty and Opportunity.

Happy is the Fund which can express its purpose in a fortunate style and title. Such a benediction is surely secured for "King George's Fund for Sailors." Our "Sailor Prince" has become our "Sailor King," and the association stirs many memories and touches all loyal hearts. Amidst the press of many duties the King has shown a sure instinct for the deeper thought and abiding purposes of his people, and has touched with a sympathetic hand those personal griefs which are in all our homes. Not on some detached height and shrouded in mystery and dignity does the nation recognise its Sovereign. On the contrary, the King is felt to be one with his subjects in the anxieties, self-denials, and dangers of the hour, strict in attention to the claims of duty, ready with helpful example, and happy in the appropriate word. Let us not forget the capacity for initiative and leadership which has shown itself to be free from the limitations of routine and ready to rise to the demands of the hour. Is it not, therefore, certain that, approved and endorsed by the King, the new Fund is happy in the circumstances of its birth?

The object of the Fund and the date of its arrival are not less charged with appeal. At no time in the nation's history has there been an occasion when sc great a stake has been committed to the care of those who go down to the sea in ships, and never has the confidence of the country in its defenders been more secure. The sea with its great traditions of enterprise and of daring is in some sense in the blood of all of us, and its large freedom and opportunity are part of our national inheritance. Its perils act only as a stimulus to courage and resolution, and deep down in the national soul is the conviction that here indeed is the element where alone can be attained the conditions of safety and of Empire. The men who face these perils and who command the e conditions have a special place in the affections of our people, and their welfare is a matter of warm-hearted concern in all our homes. There is a challenge for everyone in a Fund which announces that something remains for practical service in this direction and which affords an opportunity for the expression of national gratitude and goodwill. An appeal for our sailors cannot be made in vain, for none occupy a more secure place in the love and confidence of our people.

Were an appropriate hour needed for such an appeal it is surely here and now. In a somewhat vague, but by no means in an unreal fashion, the country is aware both of the sure protection which has been afforded by the Navy, and of the patience and hardship and danger which this protection has involved. Impatient voices have sometimes prayed for theatrical displays and hazardous enterprises, but most of us are satisfied that the Navy knows its own business, and is the best judge alike of methods and of time and the event. The mass of the people indeed take this conviction for granted, and to doubt the ability or judgment or courage of our sailors never occurs to them. They are quite aware where lies the protection of everything that is dear to them. The day will doubtless come when some of the acts and accomplishments which this protection includes will be displayed in the face of the nation, and there can be no doubt that the record will announce individual achievements equal to anything yet written on the roll of fame. The exigencies of war and the risk of giving information to the enemy compel silence about deeds which would, if revealed, electrify the nation, and with this silence we must fain strive to be content. The experience affords a special opportunity for the expression of unhesitating confidence, and an opening for this expression is now afforded in King George's Fund for Sailors.

Let it be plainly understood that the Fund intends to merit its wide and catholic title. It is not to be limited to those whose regular profession it is to direct and man our fighting ships, but is to include all of those whose business is on the great waters, for all alike serve the country and merit the nation's gratitude. Often it is said that the present war is one of nations, and not merely one of fighting men. Nowhere, so far as this country is concerned, does this saying apply more fully than to the war at sea. The Fleet nowadays includes many strange craft transferred from peaceful ends to warlike enterprises, and with these are men wise in the industries and cunning of the seas, and now set upon other than their usual spoil. Engaged in watching, patrolling, mine-sweeping, and suchlike duties are numbers of our fishermen and others skilled in seacraft, not to speak of crowds of adventurous youth who have gladly taken service in positions where danger is on every hand. Danger, however, is not alone to those who sail under the White Ensign. The piratical proceedings of the enemy spare neither combatant nor non-combatant, and our Mercantile Service now faces the hidden and treacherous submarine and the risks of death and suffering which this involves. Plain facts announce during the last few months what this means. Yet there has been no shirking. The tribute paid to the officers and men of the Merchant Service by the representatives of the Admiralty is warmly endorsed by the nation. In a very real sense there is no division between the Navy and the Merchant Service. Both are striving and enduring in similar fashion, and for one and the same patriotic end. Did circumstances permit, the country would fain recognise more fully the individual incidents which in one direction and the other have marked the course of such unfailing service. In the meantime, whatever other anxieties may oppress us, no one doubts that all our sailors are equal to their duty, and that at all costs this will be fulfilled. The new Fund bases itself upon this sense of community of service and will concern itself with the welfare both of the men of the Navy and those of the Merchant Service.

It will co-ordinate all the organisations which already exist for this end, two of the best known of these being the Dreadnought Hospital at Greenwich and the Hospital and School of Tropical Medicine at the Albert Dock. New developments and extensions will be undertaken as funds allow. Now is the opportunity for a grateful country to support a Fund which will have for its direction the devoted service of wise heads and capable administrators.

